# Proteomics Analysis of *Hydatigera taeniaeformis* Metacestode Stage

**DOI:** 10.3389/fvets.2020.00474

**Published:** 2020-08-06

**Authors:** Xiaola Guo

**Affiliations:** State Key Laboratory of Veterinary Etiological Biology, Key Laboratory of Veterinary Parasitology of Gansu Province, Lanzhou Veterinary Research Institute, CAAS, Lanzhou, China

**Keywords:** *Hydatigera taeniaeformis*, metacestode, tegument protein, Antigen B, proteome

## Abstract

*Hydatigera taeniaeformis* (*H. taeniaeformis*) is one of the most robust of tapeworm parasites that is widely distributed around the world. Information of proteins of *H. taeniaeformis* has not previously been reported. Using liquid chromatography tandem-mass spectrometry (LC–MS/MS) analysis, the proteome of *H. taeniaeformis* metacestode was profiled and a total of 408 proteins were identified. Of these, 26.5% (108/408) were annotated to be associated with metabolic pathways. Consistently, Gene Ontology analysis showed that those identified proteins were mainly classified into metabolic process of the biological processes. A set of metabolic enzymes, including Fructose-1,6-bisphosphate aldolase, enolase, Glucan phosphorylase, and phosphoenolpyruvate carboxykinase, were abundant in *H. taeniaeformis* metacestodes. In addition, some rare but interesting proteins like antigens (such as tegument protein and Antigen B) were identified. The two recombinant proteins of tegument protein and Antigen B were well-recognized by the sera from the *H. taeniaeformis-*infected mice. The *H. taeniaeformis* metacestode proteome might help to find new candidates for the immunodiagnosis and vaccine development and provide valuable information on *H. taeniaeformis* biology.

## Keypoints

- A total of 408 proteins were identified in *T. taeniaeformis* metacestodes.
- Identified proteins were classified mainly to be involved in metabolic process.
- The two recombinant proteins of tegument protein and Antigen B were well-recognized by the sera from *H. taeniaeformis-*infected mice.

## Introduction

*Hydatigera taeniaeformis* (also called *Taenia taeniaeformis*) is a parasitic helminth belonging to a family of *Taenidae*. The natural life cycle of *H. taeniaeformis* alternates between a definitive host (cats or dogs) and an intermediate host (mostly rodents and less frequently lagomorphs and humans) (1, 2). Adult tapeworms inhabit the small intestine of definitive cats or dogs and shed the terminal segments into the environment. Rodents which serve as the intermediate hosts are infected by ingesting the feed or water contaminated eggs or gravid proglottids (3, 4). Oncosphere larvae hatched from eggs in the small intestine. The larvae were passed through the digestive tract and transported passively though blood or lymph vessels to the livers, where the oncosphere larvae develop into metacestode larvae. *Cysticercus fasciolaris* is a metacestode stage of *H. taeniaeformis*. Recently, there are many reports of *H. taeniaeformis* infection in wild rodents (5–7). Our recent results highlight the prevalence of *H. taeniaeformis* in wild rodents and a risk of opportunistic parasite infection in human populations, especially those who have close contact with pets (7).

To date, proteomic studies of several cestodes have been reported, including *Echinococcus spp*. (8, 9), *Taenia solium* (10), *Taenia hydatigena* (11), and *Taenia ovis* (12). However, information of proteins of *H. taeniaeformis* has not previously been reported. This study is the first to describe the analysis of *H. taeniaeformis* metacestode protein extracts by Liquid chromatography and tandem mass spectrometry (LC-MS/MS). Identification and characterization of proteins from *H. taeniaeformis* metacestode might help to find new candidates for the immunodiagnosis and vaccine development and provide valuable information on *H. taeniaeformis* biology.

## Materials and Methods

### Parasites

By surveying on parasites in wild rodents in Xiji County, we obtained cysts in mouse livers. Fresh metacestodes were carefully dissected from the livers of naturally wild rodents as previously described (7). Combined with the morphological characteristics and host preference, the isolated parasites were suspected to be *H. taeniaeformis* (Figure S1). Then parasites were further identified using mitochondrial *cox1* genes as a molecular marker. Sequence analysis showed that the *cox1* nucleotide sequences were 1620 bp in length and shared 99% identity with that of *H. taeniaeformis*. Phylogenetic tree was constructed by the maximum likelihood method based on sequences of *mitochondrial cytochrome oxidase I* (*cox1*). In the phylogenetic tree based on *cox-1* sequences, both Miaoping (MP) and Wangping (WP) isolates were clustered together with *H. taeniaeformis* isolated from China (FJ886784, FJ597547, and NC_014768), Germany (JQ663994.1), and Japan (AB221484.1) with high probabilities (Figure S2). The dissected parasites were washed with ice-cold PBS. Afterwards, parasites were immediately frozen in liquid nitrogen and then stored at −80°C until use.

### LC–MS/MS Analysis and Protein Identification

*Hydatigera taeniaeformis* metacestodes were grounded into powder and homogenized in 100 μL of lysis buffer (containing 0.5 mM PMSF and 1 × proteinase inhibitor), and then added 6 M urea to the mixture. After centrifugation, the supernatant proteins (approximately 2 mg) were digested with 40 μL trypsin buffer (3 μg trypsin (Promega, USA) in 40 μL (25 mM) NH_4_HCO_3_) in a 37 °C water bath for 16–18 h. This digest was transferred to clean ultrafiltration tubes fitted with 10 kDa membranes, and centrifuged at 14,000 × g for 10 min. Peptide sequencing was conducted using high performance liquid chromatography coupled tandem mass spectrometry (LC-MS/MS) in Q-Exactive (Thermo Fisher Scientific) as described previously (12). The raw LC-MS/MS data was analyzed with the Mascot search engine (version 2.3) against *H. taeniaeformis* protein database retrieved from WormBase Parasite1 (http://parasite.wormbase.org/Hydatigera_taeniaeformis_prjeb534/Info/Index/) with following parameters: tryptic-specific peptides, maximum of one missed cleavages, a peptide mass tolerance of 20 ppm and a MS/MS tolerance of 0.5 Da. Label-free quantitation of proteins were calculated by intensity-based absolute quantification (iBAQ). Gene Ontology (GO) terms and Kyoto Encyclopedia of Genes and Genomes (KEGG) pathways for the identified proteins were annotated.

### Cloning, Expression, Purification, and Serological Evaluation of the Recombinant Proteins

The coding sequences of tegument protein and Antigen B genes were amplified with their specific primes. PCR fragments were digested and subcloned into prokaryotic expression plasmid pET-28a. The recombinant plasmids were transformed into *Escherichia coli* BL21 (DE3) cells. For the expression and purification of the recombinant proteins, bacteria were induced with 0.5 M isopropyl-b-D-thiogalactoside (IPTG) and the recombinant proteins were purified by Ni Sepharose 6 Fast Flow (GE Healthcare) and checked by 15% sodium dodecyl sulfate-polyacrylamide gel electrophoresis SDS-PAGE gel.

#### Western Blotting Analysis

The recombinant proteins were separated by 12% SDS-PAGE and transferred to a PVDF membranes using a Semi-Dry electrophoretic transfer cell (BioRad) for 30 min. The membranes were incubated with 5% (w/v) non-fat for 1 h and then incubated overnight at 4°C with mouse ant-sera against *H. taeniaeformis* (1:1,000, v/v dilution). Finally, they were washed and incubated with a horseradish peroxidase (HRP)-conjugated goat anti-mouse IgG (1:1,000, v/v dilution; Boster, Wuhan, China) for 1 h. Signals were visualized by exposing X-ray films using Pierce ECL Western Blotting Substrate (ThermoFisher Scientific, USA).

## Results and Discussion

**Figure 1:**
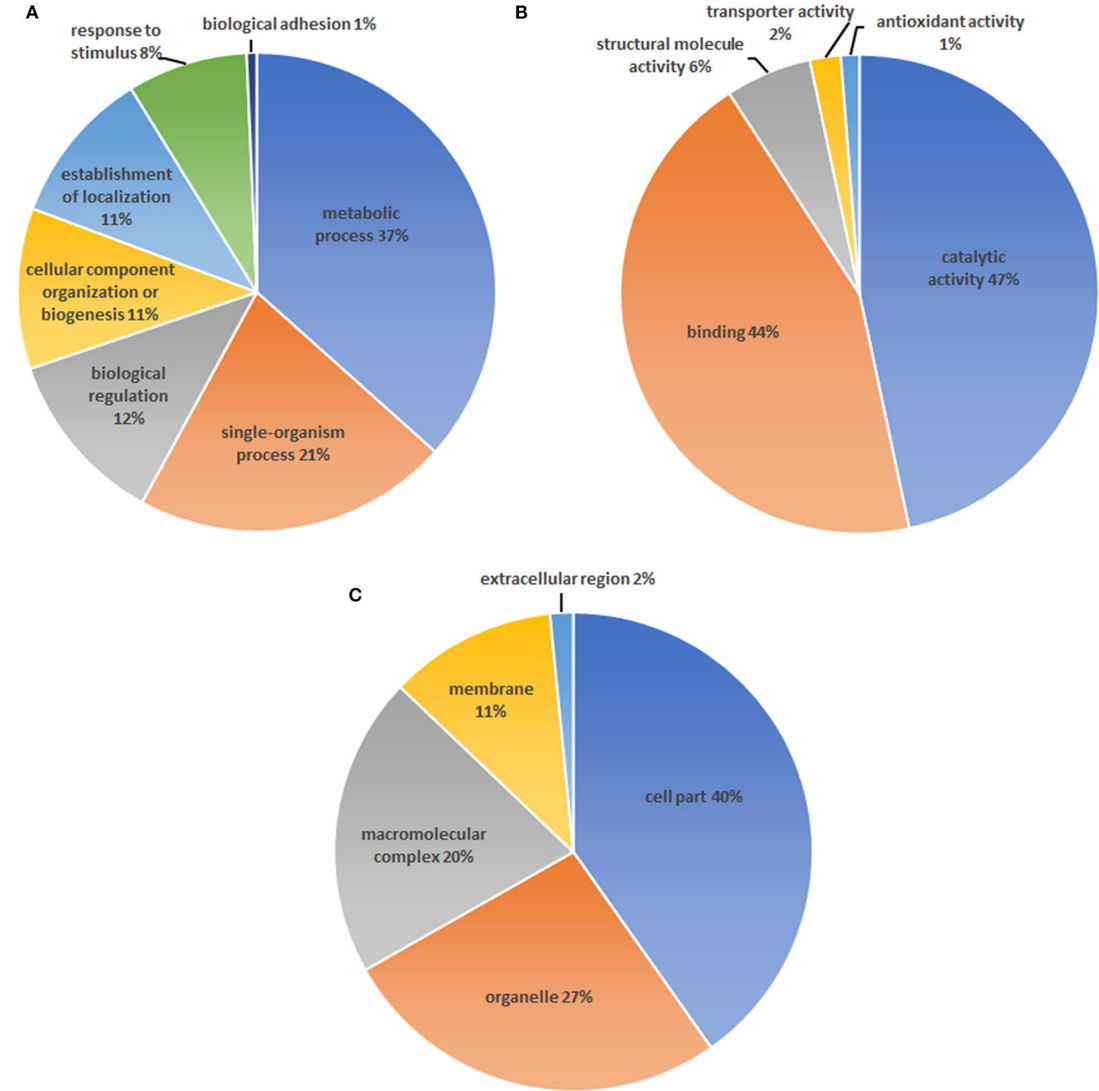
Gene ontology analysis of proteins identified in the *Hydatigera taeniaeformis* metacestodes. **(A)** biological processes. **(B)** Molecular function. **(C)** Cellular component.

**Figure 2:**
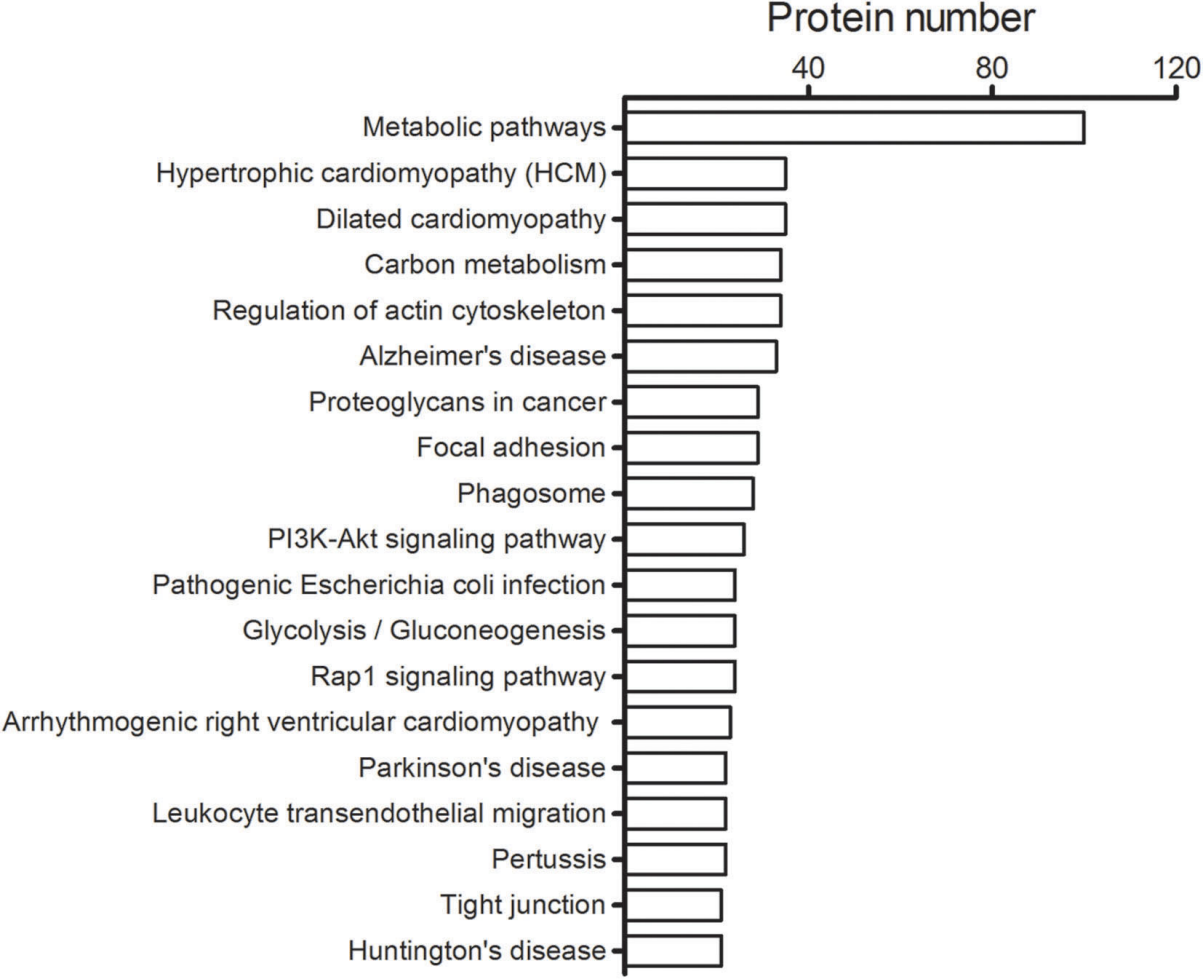
Top 20 pathways identified for the *H. taeniaeformis* metacestodes. The number of proteins involved in each of pathways is shown beside individual columns.

A total number of 4,024 spectra were detected and 2,946 unique peptides were obtained by LC-MS/MS in *H. taeniaeformis* metacestodes. In total, 408 proteins were identified by at least two unique peptides (Table S1). Similarly, 633 proteins with at least 2 unique spectra were identified from *Taenia ovis* metacestodes (12). The identified proteins include many high-abundant house-keeping proteins, such as actin, paramyosin, heat shock protein 70 (HSP70), and phosphoenolpyruvate carboxykinase, but also some rare but interesting proteins like antigens (tegument and immunogenic proteins) and receptors. The biological processes category showed enrichment of proteins involved in the metabolic process (36.6%) (Figure 1A), whereas molecular function showed a significant enrichment in catalytic activity (46.7%) and binding (44.2%) (Figure 1B). Within the cellular component category, the most enriched components were associated with structural proteins (40.2%) (Figure 1C). GO term enrichment analysis of identified proteins were shown in Figure S3. KEGG analysis showed that 408 proteins were annotated into 241 pathways, predominantly metabolic pathways (Figure 2).

According to cluster of orthologous group (COG) functional classification, 287 identified proteins were mainly grouped into six categories (metabolism, cytoskeletal proteins, post-translational modification, signal transduction, translation, and other proteins) (Table S1). Approximately, 29.7% (121 proteins) of all identified proteins could not be assigned to any functional category (annotated as uncharacterized proteins) due to many proteins whose function were uncharacterized. Most of the identified *H. taeniaeformis* proteins are related to the metabolism (Table S1). Three metabolic enzymes were most abundant in the metacestodes of *H. taeniaeformis*, including glucan phosphorylase, fructose-bisphosphate aldolase (FBA), and enolase (Table 1). Higher abundance of three metabolic enzymes suggest that the sugar metabolism is an important event in the metacestodes of *H. taeniaeformis*. Whether the high expression of fructose-bisphosphate aldolase and enolase is beneficial for parasite's growth and development needs to be investigated in future. Previous studies showed enolase has primarily functioned as glycolytic enzymes in the cytoplasm (13–15). Besides the cytoplasmic localization, enolase was detected in the tegument of protoscolexes and adult worms, suggesting potentially participated in host–parasite molecular crosstalk (13). The absence of a typical N-terminal signal peptide indicates that both FBA and enolase does not transit through the classical secretory pathway but it possibly enters an exosome-mediated secretion. Both FBA and enolase has been identified in the hydatid fluid of various helminth parasites, and excretory–secretory (ES) products and exosome-like vesicles from *in vitro*-cultured protoscoleces (13, 16–18), showing potential as antigenic candidate antigens for the diagnosis of parasitic infections.

**Table 1:** Summary of proteins identified of *Hydatigera taeniaeformis* metacestodes.

**Gene**	**Protein (name)**	**Molecular (kDa)**	**Coverage (%)**	**Number of unique peptides**	**Number of unique spectra**	**Score**	**Accession number**
**METABOLISM**							
TTAC_0000571201	Cation transport ATPase	112	0.20	33	38	676	gi|576693508
TTAC_0000058201	Glucan phosphorylase	73	0.15	22	28	634	gi|674566587
TTAC_0000683701	Fructose-1,6-bisphosphate aldolase	40	0.24	20	32	334	gi|674574479
TTAC_0001137901	Enolase	47	0.38	20	30	597	gi|563425937
TTAC_0000019601	Phosphomannomutase	69	0.13	19	27	426	gi|674571151
TTAC_0000813701	UDP-glucose pyrophosphorylase	57	0.10	17	21	496	gi|674569954
TTAC_0000696001	Glucose-6-phosphate isomerase	62	0.15	16	22	692	gi|154369446
TTAC_0000026201	1,4-alpha-glucan branching enzyme	80	0.06	15	16	749	gi|674565637
TTAC_0001048601	Glycogen debranching enzyme	190	0.06	15	15	632	gi|576699668
TTAC_0000969301	Transketolase	71	0.09	13	16	546	gi|576691476
TTAC_0000089201	6-phosphofructokinase	89	0.07	13	14	622	gi|576694014
**CYTOSKELETON**							
TTAC_0000368501	Myosin heavy chain	225	1.30	115	187	813	gi|674572847
TTAC_0001070701	Actin	42	0.87	27	58	1739	gi|576695773
TTAC_0001070701	Paramyosin	108	0.42	46	64		gi|42560539
TTAC_0000957801	Spectrin beta chain, brain 3	154	0.12	23	28	331	gi|576699919
TTAC_0000854001	Filamin-A	115	0.08	14	16	259	gi|576697079
TTAC_0000994001	tubulin alpha	50	0.09	13	17	760	gi|674570366
TTAC_0000016301	Tubulin beta	50	0.07	11	13	750	gi|29337144
TTAC_0000445201	Myophilin	21	0.11	10	13	102	gi|576693412
TTAC_0000923601	actin-binding protein	31	0.05	8	10	723	gi|576692431
**POSTTRANSLATIONAL MODIFICATION**							
TTAC_0000021301	Heat shock protein 70	71	0.44	35	55	97.23	gi|674571132
TTAC_0000230601	Molecular chaperone HtpG	79	0.12	19	24	75.86	gi|358339046
TTAC_0000028701	Rab GDP dissociation inhibitor alpha	50	0.10	16	21	98.88	gi|576694030
TTAC_0001123801	Heat shock protein 60	61	0.07	13	15	96.74	gi|674580112
TTAC_0000664001	Trypsin-like protein	54	0.15	11	16	100	gi|311335041
TTAC_0000798801	Cyclophilin	18	0.26	10	21	100	gi|589812132
TTAC_0000206801	Peroxiredoxin	22	0.20	10	15	100	gi|223403612
**SIGNAL TRANSDUCTION**							
TTAC_0000997001	14-3-3 protein	28	0.18	18	29	94.33	gi|576698574
TTAC_0001079001	Annexin A8	38	0.06	8	10	85.03	gi|576701205
TTAC_0000174201	Serine/threonine-protein phosphatase 2A	36	0.03	4	5	97.79	gi|674570693
TTAC_0000732401	Serine/threonine-protein phosphatase PP1	38	0.01	2	2	99.69	gi|576701522
**TRANSLATION**							
TTAC_0000129601	Threonyl tRNA synthetase C	83	0.08	13	15	91.54	gi|674577779
TTAC_0000647401	Elongation factor 2	99	0.12	12	16	97.29	gi|674570164
TTAC_0000304501	Histones H3 and H4	11	0.04	6	7	100	gi|602727727
TTAC_0001162901	Histone H2A	14	0.03	4	4	99.22	gi|674577655
**OTHER PROTEINS**							
TTAC_0000686001	Major vault protein	97	0.16	21	28	93.32	gi|674561667
TTAC_0000225101	P29	27	0.56	14	22	94.12	gi|558698068
TTAC_0000137701	Tegumental protein	12	0.13	8	16	95.96	gi|60459970
TTAC_0000651801	Immunogenic protein Ts76	8	0.01	2	3	100	gi|7339853
TTAC_0000705701	Antigen B	10	0.42	2	2	99.77	gi|42560539

A group of cytoskeletal proteins appeared to be very abundant in the H. taeniaeformis metacestodes stage, including actin, paramyosin, actin and related proteins (Table 1). These proteins have also been identified in protoscoleces from other tapeworm parasites like *Echinococcus multilocularis* (9). Several studies showed that helminth cytoskeletal proteins could be potential vaccine candidates (e.g., paramyosin and actin-binding protein) or targets of parasite motility (19, 20). Of them, paramyosin, a 97 kDa myofibrillar protein, can induce effective anti-infective immune protection (21). Previous study showed monoclonal anti-paramyosin antibody conferred good protection against *Schistosoma japonicum* in mice (22). A recent study showed that the recombinant paramyosin protein also conferred significant levels of protection against *S. japonicum* infection in water buffalo (19). Therefore, the high-abundant paramyosin may render it as a promising drug target or vaccine antigen against *H. taeniaeformis*. Two heat shock proteins (HSP70 and HSP60), related to posttranslational modification, were very abundant in the *H. taeniaeformis* metacestodes (Table 1). Several studies showed flatworm HSP is considered as vaccine and diagnostic targets for parasitic diseases (23–25).

Proteins of signaling transduction group were dominated by 14-3-3 and Annexin A8 (Table 1). The 14-3-3 proteins as adaptor molecular are implicated in many signaling mechanisms due to their interaction with various target proteins (26). A more extensive function of 14–3-3 proteins have been studied in *Schistosoma* spp. and *Echinococcus* spp (27–29). Multiple isoforms of 14-3-3 proteins exist in different parasitic organisms. For example, seven different 14–3-3 isoforms were found in *S. mansoni* and S. *japonicum* while five different 14-3-3 isoforms were detected in genus Echinococcus (28, 30). Interesting, 14–3-3 were detected in excretion/secretion products and exosome-like vesicles from *in vitro*-cultured protoscoleces (31). The important roles of 14–3-3 proteins in parasite proliferation and survival suggested that these proteins have potential as vaccine candidates.

**Figure 3:**
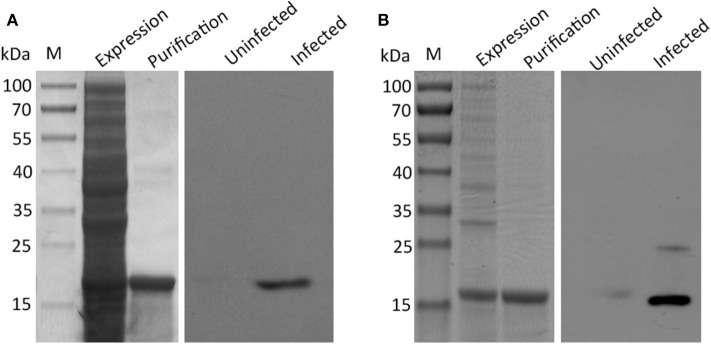
SDS-PAGE and western blot analysis of recombinant proteins of tegument protein **(A)** and Antigen B **(B)**. The expression and purification of the recombinant proteins were run on 12% SDS-PAGE, respectively. Western blot analysis of recombinant proteins using sera from *H. taeniaeformis*-infected and uninfected mice.

Three typical nuclear protein homologs grouped into translation group were identified, including an elongation factor EF-2, an adenine nucleotide translocator, and Histones H3/4. The presence of elongation factors has been detected in *Taenia saginata* and *Taenia asiatica* (32). *Echinococcus granulosus* elongation factor-1 β/δ (EgEF-1 β/δ) is an allergenic molecule of potential clinical importance of the intensity of echinococcosis immune response (33). Histones H3 have been identified as a biomarker for neoblasts in *E. multilocularis* metacestode (34). Several identified proteins were previously characterized as antigenic or involved in mechanisms of host immune evasion, such as Antigen B (AgB) (35), tegumental protein (TegP) (36), and putative major vault protein (37). Early studies have proposed that protective antibodies in serum may only be effective on the very early stages of *H. taeniaeformis* (38, 39). A successful immunization of mice against *H. taeniaeformis* using solubilized metacestode proteins suggested that more effective functional antigens appear to be present in the somatic antigens extracted from the metacestodes (40, 41). It would be interesting to screen functional antigens from the metacestodes and evaluate their protective roles against *H. taeniaeformis* infection. In present study, we selected and evaluated the immunoreactivity of two antigens (TegP and AgB) by western blot. The recombinant proteins of HtTegP and HtAtB were about 18 and 16 kDa on the SDS-PAGE, respectively (Figure 3). Western blot assays revealed that they could be reacted with mouse anti-sera against *H. taeniaeformis*, but not by the negative control serum, indicating the good immunoreactivity of recombinant proteins (Figure 3). Tegumental proteins (TegPs) are a group of proteins that coat on worm's entire outer surface (36). A growing number of studies have shown that parasite TegPs are associated with host immune responses during infection (42, 43). Recently study showed that CsTegP may be a potential druggable target (44). In *E. multilocularis*, TegP11 was primarily localized in the surface of protoscoleces and has an immunoregulatory capacity on RAW246.7 macrophages (36). Antigen B has received more attention due to its high specificity and good immunogenicity (45). Several studies showed Antigen B has great potential potentiality for diagnosis of human cystic echinococcosis (46, 47). Further studies will be evaluated for their immunodiagnostic value and immune protection against *H. taeniaeformis* infection.

## Conclusions

A total of 408 proteins were identified in *T. taeniaeformis* metacestodes, most of which were classified mainly to be involved in metabolic process. The two recombinant proteins of tegument protein and Antigen B were well-recognized by the sera from *H. taeniaeformis-*infected mice. Identification and characterization of proteins from *H. taeniaeformis* metacestode might help to find new candidates for the immunodiagnosis and vaccine development.

## Data Availability Statement

The raw data supporting the conclusions of this article will be made available by the authors, without undue reservation, to any qualified researcher.

## Ethics Statement

Animal experiments were approved by Animal Ethics Committee of Lanzhou Veterinary Research Institute, Chinese Academy of Agricultural Sciences. All animal experiments in the study were handled in strict accordance with good animal practice of Animal Ethics Procedures and Guidelines of the People's Republic of China.

## Author Contributions

XG performed study design, data analysis, and preparation of the manuscript.

### Conflict of Interest

The author declares that the research was conducted in the absence of any commercial or financial relationships that could be construed as a potential conflict of interest.
