# Supplementary material for: Proteomics Analysis of Hydatigera taeniaeformis Metacestode Stage
Source: Front Vet Sci. 2020 Aug 6;7:474. doi: 10.3389/fvets.2020.00474 (PMC7438934; doi:10.3389/fvets.2020.00474)
Supplement: Supplementary file 1 [file Presentation_1.pdf]

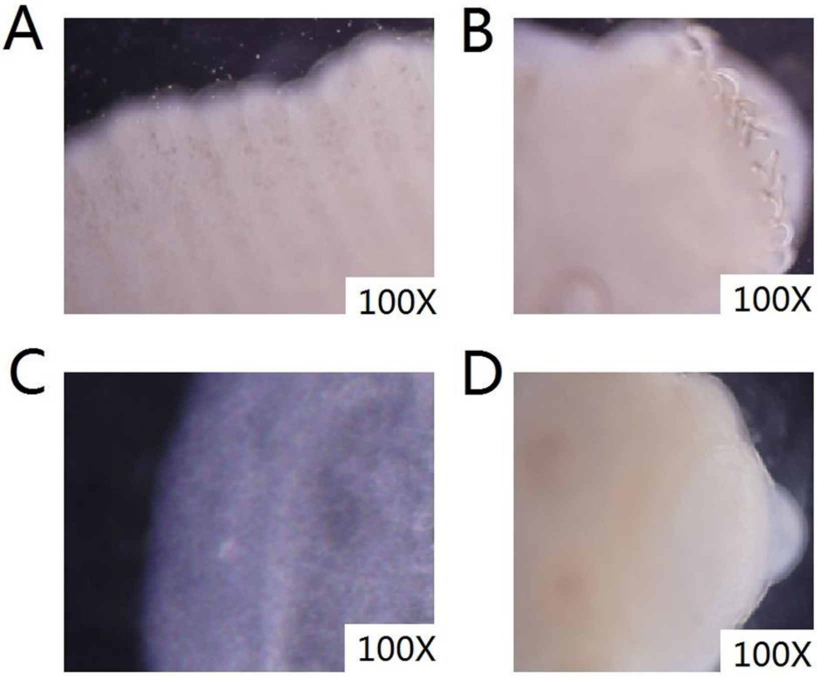

Fig. S1. The morphology of two parasites was in general similar. Both were ~ 2.5 cm in length (A) and had a large scolex with four suckers and pseudo-segmented tegument (B-D).

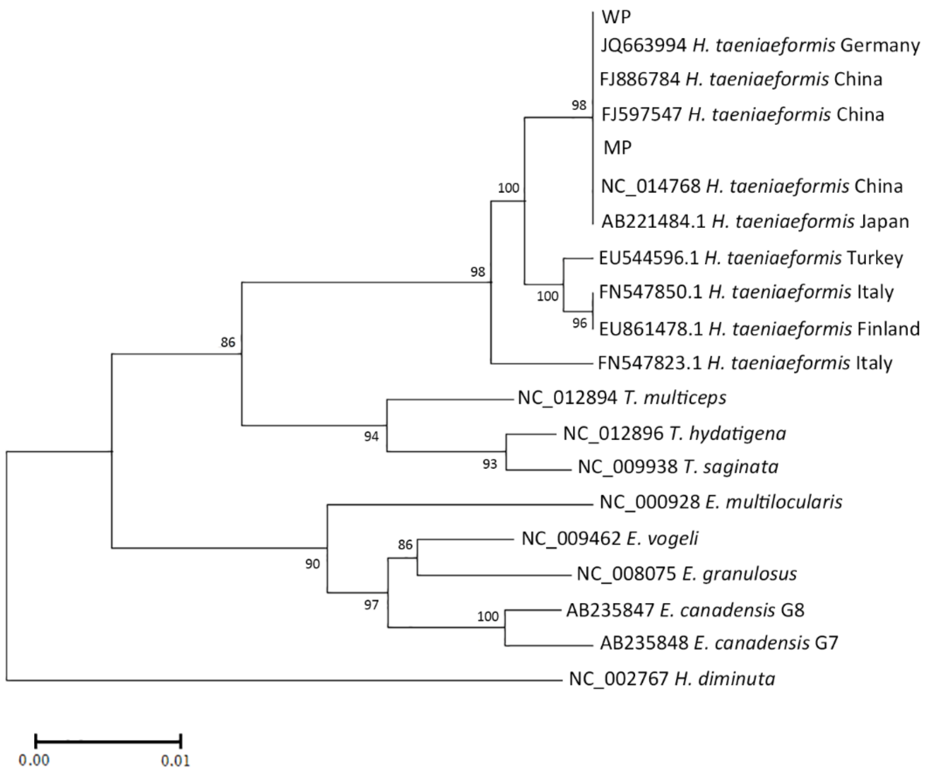

Fig. S2. Phylogenetic Tree constructed based on partial sequences of mitochondrial *cytochrome oxidase I* (*cox1*). Country of origin and isolate details are given for all *T. taeniaeformis*. GenBank accession numbers for each cox1 were shown behind '_'. Two WP and MP isolates constituted a separate clade with other isolates from China, Germany, and Japan.

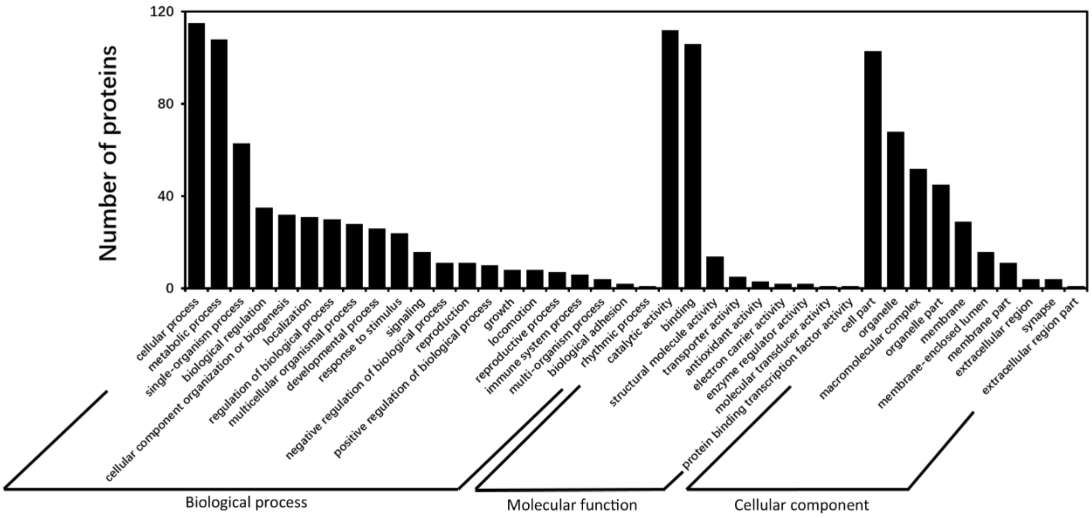

Fig. S3. Function annotations of identified proteins from *H. taeniaeformis* metacestodes.
