# Supplementary material for: Proteomics Analysis of Hydatigera taeniaeformis Metacestode Stage
Source: Front Vet Sci. 2020 Aug 6;7:474. doi: 10.3389/fvets.2020.00474 (PMC7438934; doi:10.3389/fvets.2020.00474)
Supplement: Supplementary file 2 [file Presentation_2.pdf]

Table S1. Proteins identified in *H. taeniaeformis* metacestodes.

ID	Molecular (kDa)	Percent (%)	Unique peptide	Unique Spectra	Description	COG Functional-Categories	NCBInr Accession
1	225	1.30	115	187	myosin heavy chain	Structural/cytoskeletal proteins	gi|674572847|emb|CDS41550.1|
2	108	0.42	46	64	paramyosin	-	gi|42560539|sp|P35418.2
3	882	0.27	39	46	basement membrane specific heparan sulfate	-	gi|674562108|emb|CDS23619.1|
4	71	0.44	35	55	heat shock 70 protein 4	Post-translational modification	gi|674571132|emb|CDS43123.1|
5	110	0.2	33	42	spectrin alpha actinin	-	gi|674561275|emb|CDS24366.1|
6	112	0.2	33	38	sodium/potassium-transporting ATPase subunit alpha	Metabolism	gi|576693508|gb|EUB57119.1|
7	70	0.46	30	51	phosphoenolpyruvate carboxykinase	Energy production and conversion	gi|283466482|emb|CBH36496.1|
8	42	0.87	27	58	actin	Structural/cytoskeletal proteins	gi|576695773|gb|EUB59337.1|
9	190	0.13	26	30	hemicentin 1	-	gi|674576561|emb|CDS36997.1|
10	127	0.27	26	33	type II collagen B	-	gi|674572459|emb|CDS42884.1|
11	325	0.12	25	29	titin	-	gi|674567709|emb|CDS16820.1|
12	117	0.15	25	30	spectrin beta chain brain 3	-	gi|674573973|emb|CDS40896.1|
13	72	0.23	24	34	succinate dehydrogenase flavoprotein subunit	Energy production and conversion	gi|576699397|gb|EUB62925.1|
14	37	0.67	24	43	cytosolic malate dehydrogenase	Energy production and conversion	gi|323361126|gb|ADX42057.1|
15	154	0.12	23	28	Spectrin beta chain brain 3	Structural/cytoskeletal proteins	gi|576699919|gb|EUB63443.1|
16	73	0.15	22	28	glycogen phosphorylase	Metabolism	gi|674566587|emb|CDS19687.1|
17	97	0.16	21	28	major vault protein	-	gi|674561667|emb|CDS23982.1|
18	42	0.18	21	26	L-threonine 3-dehydrogenase	Metabolism	gi|576693761|gb|EUB57365.1|
19	166	0.19	20	27	collagen alpha 1V chain	-	gi|674265696|emb|CDI98199.1|
20	184	0.082	20	21	clathrin heavy chain	-	gi|674267057|emb|CDI97445.1|
21	47	0.38	20	30	enolase	Metabolism	gi|563425937|gb|AHB59732.1|
22	40	0.24	20	32	fructose 16 bisphosphate aldolase	Metabolism	gi|674574479|emb|CDS41408.1|
23	28	0.16	19	27	putative growth regulator 14-3-3	Signal transduction	gi|563425939|gb|AHB59733.1|
24	166	0.086	19	21	titin	-	gi|576699428|gb|EUB62955.1|
25	79	0.12	19	24	molecular chaperone HtpG	Post-translational modification	gi|358339046|dbj|GAA47176.1|
26	60	0.1	19	19	ATP synthase subunit alpha mitochondrial	Energy production and conversion	gi|674562214|emb|CDS23514.1|
27	69	0.13	19	27	phosphoglucomutase 2	Metabolism	gi|674571151|emb|CDS43145.1|
28	183	0.12	18	20	filamin	Structural/cytoskeletal proteins	gi|674570113|emb|CDS16181.1|
29	33	0.1	18	21	tropomyosin 2 high molecular weight isoform	-	gi|29337029|sp|Q95PU1.1|

30	56	0.1	18	22	ATP synthase subunit beta	Energy production and conversion	gi|576697808|gb|EUB61347.1|
31	93	0.17	18	25	calpain-A	-	gi|576700942|gb|EUB64462.1|
32	28	0.18	18	29	14-3-3 protein zeta	Signal transduction	gi|576698574|gb|EUB62107.1|
33	57	0.1	17	21	UTP glucose 1 phosphate uridylyltransferase	Metabolism	gi|674569954|emb|CDS16022.1|
34	69	0.1	17	21	four and A half lim domains	-	gi|961439404|emb|CUT98901.1|
35	232	0.12	17	22	collagen alpha 1(IV) chain	-	gi|674561688|emb|CDS24003.1|
36	65	0.15	17	26	NADP dependent malic enzyme	Energy production and conversion	gi|674565192|emb|CDS20746.1|
37	62	0.15	16	22	phosphoglucose isomerase	Metabolism	gi|154369446|gb|ABS81352.1|
38	131	0.15	16	21	fibrillar collagen chain FAp1 alpha	-	gi|674568718|emb|CDS17836.1|
39	50	0.097	16	21	Rab GDP dissociation inhibitor alpha	Post-translational modification	gi|576694030|gb|EUB57627.1|
40	162	0.097	16	18	titin	Signal transduction	gi|674567709|emb|CDS16820.1|
41	22	0.13	16	20	myosin regulatory light chain	Structural/cytoskeletal proteins	gi|124783553|gb|ABN14929.1|
42	190	0.063	15	15	glycogen debranching enzyme	Metabolism	gi|576699668|gb|EUB63194.1|
43	80	0.063	15	16	glucan 14 alpha branching enzyme 1	Metabolism	gi|674565637|emb|CDS20187.1|
44	27	0.56	14	22	P29	-	gi|558698068|gb|AHA85391.1|
45	115	0.078	14	16	Filamin-A	Structural/cytoskeletal proteins	gi|576697079|gb|EUB60625.1|
46	65	0.13	14	21	Phosphoglucomutase	Metabolism	gi|576695399|gb|EUB58969.1|
47	118	0.074	13	13	calcium transporting atpase	Metabolism	gi|674568220|emb|CDS17335.1|
48	93	0.097	13	18	Basement membrane-specific heparan sulfate proteoglycan core protein	-	gi|576697991|gb|EUB61528.1|
49	50	0.093	13	17	tubulin alpha chain	Structural/cytoskeletal proteins	gi|674570366|emb|CDS16437.1|
50	176	0.093	13	16	abnormal EMBroygenesis family member emb 9	-	gi|674561689|emb|CDS24004.1|
51	61	0.074	13	15	heat shock protein 60	Post-translational modification	gi|674580112|emb|CDS35950.1|
52	127	0.13	13	20	Collagen alpha-2(I) chain	-	gi|576700155|gb|EUB63678.1|
53	34	0.089	13	17	atp:adp antiporter	-	gi|961439544|emb|CUT99040.1|
54	71	0.089	13	16	Transketolase	Metabolism	gi|576691476|gb|EUB55174.1|
55	83	0.082	13	15	Threonyl tRNA synthetase C	Transcription	gi|674577779|emb|CDS36622.1|
56	34	0.11	13	15	aldo keto reductase family 1 member B4	Function unknown	gi|674266307|emb|CDI97754.1|
57	89	0.071	13	14	6-phosphofructokinase	Metabolism	gi|576694014|gb|EUB57612.1|
58	17	0.25	12	19	Myosin essential light chain, striated adductor muscle	Structural/cytoskeletal proteins	gi|576700259|gb|EUB63782.1|
59	114	0.067	12	13	Ubiquitin-like modifier-activating enzyme	Metabolism	gi|576696184|gb|EUB59741.1|
60	66	0.063	12	14	NADP-dependent malic enzyme	Energy production and conversion	gi|576699687|gb|EUB63213.1|
61	42	0.11	12	16	Phosphoglycerate kinase	Metabolism	gi|576695835|gb|EUB59398.1|

62	99	0.12	12	16	elongation factor 2	Transcription	gi|674570164|emb|CDS16232.1|
63	130	0.082	12	15	filamin	-	gi|961440428|emb|CDS40988.2|
64	60	0.067	12	15	glucose 6 phosphate 1 dehydrogenase	Metabolism	gi|674564077|emb|CDS21761.1|
65	42	0.089	12	17	actin modulator protein Taenia asiatica]	-	gi|124783834|gb|ABN14949.1|
66	123	0.12	11	16	collagen type i ii iii v xi alpha	-	gi|674563099|emb|CDS22734.1|
67	54	0.15	11	16	trypsin-like protein	Post-translational modification	gi|311335041|gb|ADP89566.1|
68	111	0.063	11	13	hemicentin 1	-	gi|674576560|emb|CDS36996.1|
69	343	0.052	11	12	WD repeat-containing protein	Function unknown	gi|576693505|gb|EUB57116.1|
70	48	0.063	11	13	Adenylosuccinate synthetase isozyme	Metabolism	gi|576698414|gb|EUB61948.1|
71	46	0.063	11	15	2 amino 3 ketobutyrate coenzyme A ligase	Metabolism	gi|674266918|emb|CDI97306.1|
72	95	0.14	11	19	fasciclin-1	-	gi|576902497|gb|AHH45014.1|
73	33	0.067	11	12	Succinate dehydrogenase ubiquinone] iron-sulfur subunit	Energy production and conversion	gi|576692886|gb|EUB56517.1|
74	36	0.17	11	17	gyceraldehyde-3-phosphate dehydrogenase	Metabolism	gi|149364041|gb|ABR24229.1|
75	85	0.059	11	12	calpain	-	gi|674574770|emb|CDS39654.1|
76	50	0.074	11	13	Tubulin beta-2 chain	Structural/cytoskeletal proteins	gi|29337144|sp|Q9NFZ6.1|
77	52	0.074	11	12	adenosylhomocysteinase	Metabolism	gi|674563544|emb|CDS21893.1|
78	50	0.082	11	15	cat eye syndrome critical region protein 5	Metabolism	gi|674569255|emb|CDS15318.1|
79	80	0.067	11	14	glycogen synthase	Structural/cytoskeletal proteins	gi|674572158|emb|CDS42583.1|
80	18	0.26	10	21	cyclophilin	Post-translational modification	gi|589812132|gb|AHL20246.1|
81	39	0.071	10	14	Pyruvate dehydrogenase E1 component subunit beta	Energy production and conversion	gi|576699464|gb|EUB62991.1|
82	13	0.093	10	12	transforming growth factor beta 1 induced	-	gi|961440199|emb|CDI97591.2|
83	22	0.2	10	15	2-cys peroxiredoxin	Post-translational modification	gi|223403612|gb|ACM89281.1|
84	62	0.093	10	12	fumarate hydratase class I	Energy production and conversion	gi|674565797|emb|CDS20347.1|
85	21	0.11	10	13	Myophilin	Structural/cytoskeletal proteins	gi|576693412|gb|EUB57026.1|
86	45	0.082	10	14	Ndr	-	gi|674572505|emb|CDS42931.1|
87	75	0.067	10	11	Arginyl-tRNA synthetase, cytoplasmic	Transcription	gi|576694953|gb|EUB58531.1|
88	32	0.052	10	11	tropomyosin 2 high molecular weight isoform	-	gi|311992220|gb|ADQ26722.1|
89	88	0.033	9	9	transitional endoplasmic reticulum atpase	Post-translational modification	gi|674566315|emb|CDS19413.1|
90	69	0.048	9	11	actin interacting protein 1	Function unknown	gi|674571818|emb|CDS42236.1|
91	39	0.063	9	14	guanine nucleotide binding protein subunit	Function unknown	gi|674265669|emb|CDI98172.1|
92	46	0.086	9	12	hypothetical transcript Hymenolepis microstoma]	-	gi|961496852|emb|CUU99433.1|
93	17	0.067	9	11	Cofilin/actin-depolymerizing factor	-	gi|576697710|gb|EUB61250.1|

94	152	0.063	9	11	Peroxidasin	Function unknown	gi|576695249|gb|EUB58822.1|
95	67	0.059	9	10	glycogen phosphorylase	Metabolism	gi|674566586|emb|CDS19686.1|
96	31	0.041	9	11	Four and a half LIM domains-containing protein	-	gi|576696870|gb|EUB60419.1|
97	26	0.071	9	12	Triosephosphate isomerase	Metabolism	gi|38258647|sp|Q9GTX8.1|
98	14	0.063	9	12	Four and a half LIM domains-containing protein	-	gi|576697637|gb|EUB61178.1|
99	29	0.074	9	13	phosphoglycerate mutase	Metabolism	gi|674573499|emb|CDS40416.1|
100	70	0.048	9	10	heat shock protein 70	Post-translational modification	gi|674568757|emb|CDS17875.1|
101	192	0.052	9	11	putative ankyrin 2,3/unc44 Schistosoma mansoni]	Function unknown	gi|353232760|emb|CCD80115.1|
102	31	0.052	8	10	Actin-1	Structural/cytoskeletal proteins	gi|576692431|gb|EUB56079.1|
103	50	0.063	8	11	calponin-like protein	Structural/cytoskeletal proteins	gi|124784064|gb|ABN14962.1|
104	35	0.11	8	12	lactate dehydrogenase A	Energy production and conversion	gi|318054471|gb|ADV35656.1|
105	232	0.033	8	8	myosin heavy chain non muscle	Structural/cytoskeletal proteins	gi|674566383|emb|CDS19483.1|
106	43	0.033	8	9	Pyruvate dehydrogenase E1 component subunit alpha type I	Energy production and conversion	gi|576694194|gb|EUB57787.1|
107	29	0.037	8	8	14-3-3 protein zeta	Signal transduction	gi|576698383|gb|EUB61917.1|
108	12	0.13	8	16	tegumental protein	-	gi|60459970|gb|AAX20156.1|
109	50	0.052	8	10	dihydrolipoamide dehydrogenase	Energy production and conversion	gi|674570213|emb|CDS16281.1|
110	165	0.074	8	11	collagen type XI alpha 2	-	gi|674564565|emb|CDS20882.1|
111	38	0.063	8	10	Annexin A8	-	gi|576701205|gb|EUB64725.1|
112	58	0.052	8	10	glutamate dehydrogenase	Metabolism	gi|961439347|emb|CUT98844.1|
113	35	0.037	8	8	mitochondrial phosphate carrier protein precursor	-	gi|124782844|gb|ABN14885.1|
114	104	0.037	7	8	Alpha-actinin, sarcomeric	Structural/cytoskeletal proteins	gi|576699978|gb|EUB63502.1|
115	42	0.089	7	11	Beta-ureidopropionase	Function unknown	gi|576700488|gb|EUB64010.1|
116	135	0.026	7	7	importin 5	-	gi|674267405|emb|CDI96909.1|
117	128	0.026	7	7	Tetracycline resistance leader peptide TetL	Metabolism	gi|674580117|emb|CDS35955.1|
118	32	0.041	7	8	prohibitin	Post-translational modification	gi|674576684|emb|CDS37121.1|
119	49	0.059	7	9	elongation factor 1 alpha	Transcription	gi|815798391|gb|AKE48498.1|
120	29	0.048	7	8	14-3-3 protein zeta	Signal transduction	gi|576699818|gb|EUB63343.1|
121	42	0.074	7	11	Actin, cytoplasmic 2	Structural/cytoskeletal proteins	gi|576693212|gb|EUB56832.1|
122	63	0.041	7	8	NADP dependent malic enzyme	Energy production and conversion	gi|674562690|emb|CDS22946.1|
123	37	0.052	7	8	hemicentin 1	-	gi|674576560|emb|CDS36996.1|

124	42	0.033	7	8	cAMP-dependent protein kinase type II regulatory subunit	Signal transduction	gi|576701554|gb|EUB65073.1|
125	48	0.048	7	7	Dihydrolipoyllysine-residue acetyltransferase component of pyruvate dehydrogenase	Energy production and conversion	gi|576691716|gb|EUB55398.1|
126	66	0.086	6	11	fasciclin-2	-	gi|576902499|gb|AHH45015.1|
127	60	0.022	6	6	T complex protein 1 subunit alpha	Post-translational modification	gi|674573749|emb|CDS40669.1|
128	97	0.03	6	6	dynamin	Function unknown	gi|674266904|emb|CDI97292.1|
129	35	0.03	6	7	SH2 motif	-	gi|674563259|emb|CDS22222.1|
130	57	0.022	6	6	T-complex protein 1 subunit delta	Post-translational modification	gi|576700958|gb|EUB64478.1|
131	26	0.12	6	10	glutathione S-transferase	Post-translational modification	gi|21591409|gb|AAM64045.1|
132	16	0.033	6	6	Actin-depolymerizing factor 2	-	gi|576697711|gb|EUB61251.1|
133	30	0.033	6	8	thymidine phosphorylase	Metabolism	gi|961440116|emb|CUT99611.1|
134	11	0.093	6	12	dynein light chain	-	gi|29467010|dbj|BAC66949.1|
135	33	0.052	6	9	Calcium binding protein	Structural/cytoskeletal proteins	gi|674562291|emb|CDS23332.1|
136	36	0.056	6	8	small heat-shock protein	-	gi|21665905|emb|CAD36617.1|
137	32	0.03	6	7	Mitochondrial dicarboxylate carrier	-	gi|576692289|gb|EUB55943.1|
138	62	0.037	6	7	Asparaginyl-tRNA synthetase, cytoplasmic	Transcription	gi|576690884|gb|EUB54629.1|
139	18	0.033	6	7	Ubiquitin-conjugating enzyme E2 N	Post-translational modification	gi|576696369|gb|EUB59924.1|
140	20	0.041	6	7	glycogen phosphorylase	Metabolism	gi|674566587|emb|CDS19687.1|
141	30	0.074	6	9	purine nucleoside phosphorylase	Metabolism	gi|674567224|emb|CDS18572.1|
142	27	0.03	6	6	subfamily T1A non peptidase ue	Post-translational modification	gi|674563031|emb|CDS22665.1|
143	42	0.022	6	6	casein kinase ii subunit alpha	Signal transduction	gi|674576896|emb|CDS37336.1|
144	26	0.03	6	7	thioredoxin dependent peroxide reductase	Post-translational modification	gi|674561357|emb|CDS24295.1|
145	11	0.041	6	7	histone H3	Transcription	gi|602727727|ref|XP_007449308.1
146	22	0.041	6	8	adenylate kinase	Metabolism	gi|674563965|emb|CDS21649.1|
147	58	0.033	6	8	chaperonin containing TCP1 subunit 2 beta	Post-translational modification	gi|674574995|emb|CDS39497.1|
148	65	0.033	6	6	dihydropyrimidinase	Metabolism	gi|674567717|emb|CDS16828.1|
149	28	0.045	6	8	3 oxoacyl acyl carrier protein reductase	Metabolism	gi|674573436|emb|CDS40352.1|
150	28	0.03	6	7	Proteasome subunit alpha type-3	Post-translational modification	gi|576697420|gb|EUB60963.1|
151	44	0.03	5	6	DnaJ subfamily A member	Post-translational modification	gi|576693390|gb|EUB57005.1|
152	80	0.045	5	7	Titin	-	gi|576695343|gb|EUB58914.1|
153	82	0.019	5	5	FERM domain-containing protein 3	-	gi|576701126|gb|EUB64646.1|
154	68	0.041	5	6	H17g protein, tegumental antigen	-	gi|34368418|emb|CAE46111.1|
155	35	0.033	5	6	Putative deoxyribose-phosphate aldolase	Metabolism	gi|576695900|gb|EUB59462.1|
156	75	0.022	5	5	thioredoxin glutathione reductase	Energy production and conversion	gi|29825896|gb|AAN63052.1|

157	59	0.026	5	6	T-complex protein 1 subunit gamma	Post-translational modification	gi|674570231|emb|CDS16299.1|
158	355	0.019	5	5	Titin	-	gi|576698310|gb|EUB61845.1|
159	37	0.03	5	6	Calumenin-B	-	gi|576701509|gb|EUB65028.1|
160	115	0.03	5	5	alpha actinin sarcomeric	Structural/cytoskeletal proteins	gi|674562490|emb|CDS23200.1|
161	57	0.019	5	5	aminopeptidase N	Metabolism	gi|674572490|emb|CDS42915.1|
162	40	0.033	5	6	Succinyl-CoA ligase GDP-forming] subunit alpha	Energy production and conversion	gi|576693072|gb|EUB56696.1|
163	45	0.033	5	6	Aspartate aminotransferase	Metabolism	gi|576693792|gb|EUB57395.1|
164	14	0.048	5	7	histone H2B	-	gi|674577890|emb|CDS36286.1|
165	60	0.022	5	5	T-complex protein 1 subunit theta	Post-translational modification	gi|576696899|gb|EUB60447.1|
166	30	0.026	5	5	phosphatase 2a inhibitor i2pp2a	-	gi|674566256|emb|CDS19354.1|
167	26	0.033	5	6	Proteasome subunit alpha type-2	Post-translational modification	gi|576695969|gb|EUB59529.1|
168	42	0.033	5	7	heat shock protein 71 protein	Post-translational modification	gi|674560532|emb|CDS25069.1|
169	35	0.037	5	6	GDP L fucose synthase	Metabolism	gi|674577076|emb|CDS37519.1|
170	309	0.019	5	5	Titin	Signal transduction	gi|576698310|gb|EUB61845.1|
171	51	0.033	5	7	Innexin unc-9	-	gi|576699141|gb|EUB62670.1|
172	72	0.026	5	6	glucose-regulated protein	Post-translational modification	gi|576694898|gb|EUB58477.1|
173	53	0.019	5	5	mitochondrial processing peptidase beta subunit	Function unknown	gi|674266979|emb|CDI97367.1|
174	36	0.022	5	5	lactate dehydrogenase B	Energy production and conversion	gi|318054473|gb|ADV35657.1|
175	21	0.037	5	7	PDZ and LIM domain protein	-	gi|576695629|gb|EUB59195.1|
176	41	0.041	5	6	annexin B2	-	gi|62868630|gb|AAY17503.1|
177	11	0.086	5	8	Dynein light chain 2	-	gi|576691998|gb|EUB55665.1|
178	342	0.03	5	5	Lipid transport protein N terminal	-	gi|674562598|emb|CDS22854.1|
179	15	0.022	5	5	cytosolic fatty acid binding protein	-	gi|82412213|gb|ABB76135.1|
180	163	0.022	5	5	Lethal(2) giant larvae protein	-	gi|576692591|gb|EUB56232.1|
181	57	0.026	5	6	lysyl oxidase	-	gi|674265829|emb|CDI98333.1|
182	88	0.03	5	6	n acetylated alpha linked acidic dipeptidase 2	-	gi|674574508|emb|CDS41437.1|
183	21	0.026	5	5	ADP-ribosylation factor	Function unknown	gi|576696292|gb|EUB59848.1|
184	13	0.048	5	7	transforming growth factor beta 1 induced	-	gi|961440199|emb|CDI97591.2|
185	29	0.019	5	5	peptidase inhibitor 16	-	gi|674573188|emb|CDS40101.1|
186	45	0.019	4	4	eukaryotic initiation factor 4A	Transcription	gi|674561290|emb|CDS24381.1|
187	31	0.033	4	5	carbonyl reductase 1	Metabolism	gi|674266977|emb|CDI97365.1|
188	14	0.026	4	4	histone H2A	Transcription	gi|674577655|emb|CDS36693.1|
189	11	0.026	4	7	Dynein light chain LC6, flagellar outer arm	-	gi|576691990|gb|EUB55657.1|

190	40	0.041	4	7	calponin-like protein Taenia asiatica]	Structural/cytoskeletal proteins	gi|124783983|gb|ABN14959.1|
191	48	0.019	4	5	hypothetical protein EGR_01765	-	gi|576699749|gb|EUB63274.1|
192	41	0.037	4	7	Serpin B9	Post-translational modification	gi|576696703|gb|EUB60253.1|
193	19	0.022	4	5	titin	-	gi|674566990|emb|CDS18337.1|
194	50	0.019	4	4	Tubulin beta-1 chain	Structural/cytoskeletal proteins	gi|576697601|gb|EUB61142.1|
195	16	0.03	4	5	Chain A	Metabolism	gi|347948498|pdb|3MND|A
196	41	0.022	4	5	Calcium-binding protein 39-like protein	-	gi|576697595|gb|EUB61136.1|
197	36	0.037	4	5	transaldolase	Metabolism	gi|674560699|emb|CDS24943.1|
198	42	0.067	4	7	Severin	-	gi|576693443|gb|EUB57056.1|
199	36	0.022	4	5	Annexin A13	-	gi|576701204|gb|EUB64724.1|
200	179	0.015	4	4	Ankyrin-2	Function unknown	gi|576699604|gb|EUB63130.1|
201	31	0.022	4	4	prohibitin protein WPH	Post-translational modification	gi|674574612|emb|CDS39840.1|
202	36	0.026	4	5	serine:threonine protein phosphatase 2A	Signal transduction	gi|674570693|emb|CDS43313.1|
203	195	0.015	4	4	Muscle M-line assembly protein unc-89	-	gi|576698306|gb|EUB61841.1|
204	20	0.045	4	5	protein DJ 1	Function unknown	gi|674266694|emb|CDI97572.1|
205	15	0.022	4	5	Ubiquitin	Post-translational modification	gi|576701155|gb|EUB64675.1|
206	11	0.063	4	9	expressed protein	-	gi|674574827|emb|CDS39329.1|
207	27	0.037	4	5	ornithine aminotransferase	Metabolism	gi|674572180|emb|CDS42605.1|
208	36	0.033	4	6	NADH-cytochrome b5 reductase 3	Metabolism	gi|576700067|gb|EUB63591.1|
209	29	0.026	4	6	sj ts4 protein	-	gi|674577718|emb|CDS36766.1|
210	31	0.019	4	4	Microtubule associated protein RP:EB family	Structural/cytoskeletal proteins	gi|961439806|emb|CUT99302.1|
211	54	0.015	4	4	tubulin alpha 1C chain	Structural/cytoskeletal proteins	gi|674577457|emb|CDS36904.1|
212	29	0.019	4	5	putative cyclin-H	Post-translational modification	gi|576697794|gb|EUB61333.1|
213	30	0.022	4	5	Proteasome subunit alpha type-7-like protein	Post-translational modification	gi|576696890|gb|EUB60438.1|
214	104	0.015	4	4	multivalent antigen sj tpi	-	gi|674565534|emb|CDS20084.1|
215	218	0.019	4	4	ankyrin 23:unc44	Function unknown	gi|674566273|emb|CDS19371.1|
216	25	0.052	4	7	glutathione transferase	Post-translational modification	gi|1004227|emb|CAA59739.1|
217	9	0.026	4	5	LIM zinc bindingdomain containing protein	-	gi|674576398|emb|CDS38102.1|
218	15	0.026	4	4	histone	Transcription	gi|674569431|emb|CDS15495.1|
219	12	0.033	4	5	thioredoxin-1	Post-translational modification	gi|724471034|gb|AIY26017.1|
220	18	0.03	4	4	Tubulin polymerization-promoting protein	-	gi|576692624|gb|EUB56264.1|
221	32	0.015	4	4	GDP-mannose 4,6 dehydratase	Structural/cytoskeletal proteins	gi|576691516|gb|EUB55210.1|
222	52	0.019	4	4	Aldehyde dehydrogenase family 1 member A3	Energy production and conversion	gi|576693855|gb|EUB57457.1|

223	52	0.015	4	4	NADH dehydrogenase ubiquinone flavoprotein 1	Energy production and conversion	gi|674578154|emb|CDS36554.1|
224	20	0.03	4	7	cysteine and glycine rich protein 1	-	gi|674574369|emb|CDS41298.1|
225	52	0.019	4	5	hexokinase	Metabolism	gi|674576035|emb|CDS38325.1|
226	66	0.019	4	4	Phosphoacetylglucosamine mutase	Metabolism	gi|576698281|gb|EUB61816.1|
227	42	0.011	3	3	beta centractin	Structural/cytoskeletal proteins	gi|674568090|emb|CDS17204.1|
228	38	0.015	3	4	26S proteasome non ATPase regulatory subunit 7	Function unknown	gi|674574868|emb|CDS39370.1|
229	19	0.015	3	4	Calmodulin	Structural/cytoskeletal proteins	gi|576691891|gb|EUB55563.1|
230	21	0.015	3	3	ribosomal protein L11	Transcription	gi|674266506|emb|CDI97959.1|
231	39	0.015	3	3	Guanine nucleotide-binding protein subunit beta	Function unknown	gi|576698293|gb|EUB61828.1|
232	25	0.011	3	3	fascin 2	-	gi|674564399|emb|CDS21401.1|
233	34	0.019	3	4	2-oxoglutarate/malate carrier protein	-	gi|576692217|gb|EUB55875.1|
234	49	0.011	3	3	spliceosome rna helicase bat1	Transcription	gi|674564743|emb|CDS21061.1|
235	19	0.026	3	4	aldo keto reductase family 1 member B4	Function unknown	gi|674564193|emb|CDS21191.1|
236	56	0.019	3	3	alanine aminotransferase 2	Metabolism	gi|674566348|emb|CDS19447.1|
237	25	0.011	3	3	platelet activating factor acetylhydrolase IB	-	gi|674267045|emb|CDI97433.1|
238	33	0.019	3	4	inorganic pyrophosphatase	Energy production and conversion	gi|674577167|emb|CDS37611.1|
239	10	0.026	3	5	Dynein light chain LC6, flagellar outer arm	-	gi|576695376|gb|EUB58946.1|
240	47	0.015	3	3	NADH dehydrogenase ubiquinone Fe S protein 1	Energy production and conversion	gi|674568845|emb|CDS17967.1|
241	31	0.015	3	3	adenylate cyclase 9	-	gi|674571922|emb|CDS42344.1|
242	21	0.011	3	3	ras protein Rab 7a	Function unknown	gi|674266074|emb|CDI98578.1|
243	51	0.019	3	4	Tubulin beta-3 chain	Structural/cytoskeletal proteins	gi|576697602|gb|EUB61143.1|
244	122	0.011	3	3	Anoctamin-1	-	gi|576691278|gb|EUB54990.1|
245	42	0.011	3	3	NAD(P) transhydrogenase subunit alpha	Energy production and conversion	gi|576694192|gb|EUB57785.1|
246	50	0.019	3	3	Non-lysosomal glucosylceramidase	Metabolism	gi|576694850|gb|EUB58430.1|
247	74	0.015	3	4	hypothetical protein EGR_00474	-	gi|576701004|gb|EUB64524.1|
248	28	0.011	3	3	NADH dehydrogenase ubiquinone iron sulfur	Energy production and conversion	gi|961440051|emb|CUT99546.1|
249	57	0.011	3	3	coronin 1C	Function unknown	gi|674266210|emb|CDI98717.1|
250	61	0.019	3	5	pyruvate kinase	Metabolism	gi|674572621|emb|CDS43052.1|
251	53	0.011	3	3	4-hydroxybutyrate coenzyme A transferase	Energy production and conversion	gi|576692646|gb|EUB56285.1|
252	53	0.015	3	3	Cytochrome b c1 complex subunit 2	Function unknown	gi|674562375|emb|CDS23417.1|
253	22	0.019	3	4	rho gdp dissociation inhibitor	-	gi|674571096|emb|CDS43731.1|
254	24	0.03	3	3	sigma-type glutathione S-transferase	-	gi|225355240|gb|ACN88552.1|
255	20	0.015	3	4	translationally controlled tumor protein	-	gi|674267348|emb|CDI96852.1|
256	62	0.011	3	3	glycerol kinase	Energy production and conversion	gi|674266527|emb|CDI97981.1|

257	40	0.011	3	3	stomatin protein 2	Post-translational modification	gi|674571420|emb|CDS41834.1|
258	21	0.011	3	3	NADH dehydrogenase ubiquinone] 1 alpha subcomplex subunit	-	gi|576695550|gb|EUB59117.1|
259	26	0.015	3	3	Synaptic vesicle membrane protein VAT-1-like protein	Energy production and conversion	gi|576698237|gb|EUB61772.1|
260	38	0.011	3	3	alpha tocopherol transfer protein	-	gi|674562106|emb|CDS23617.1|
261	102	0.019	3	3	Lysosomal alpha-glucosidase	Metabolism	gi|576693012|gb|EUB56638.1|
262	10	0.045	3	6	Dynein light chain 1	-	gi|576698380|gb|EUB61914.1|
263	54	0.011	3	3	6-phosphogluconate dehydrogenase	Metabolism	gi|576699896|gb|EUB63420.1|
264	63	0.011	3	3	non lysosomal glucosylceramidase	Metabolism	gi|674570277|emb|CDS16346.1|
265	31	0.011	3	3	fructose-1,6-phosphatase	Metabolism	gi|338827796|gb|AEJ15818.1|
266	75	0.011	3	3	ubiquitin protein ligase BRE1	Structural/cytoskeletal proteins	gi|674569884|emb|CDS15952.1|
267	58	0.015	3	3	Aspartyl-tRNA synthetase, cytoplasmic	Transcription	gi|576701543|gb|EUB65062.1|
268	27	0.011	3	3	Proteasome subunit alpha type-6	Post-translational modification	gi|576694857|gb|EUB58437.1|
269	26	0.015	3	3	60S ribosomal protein L10a	Transcription	gi|674569308|emb|CDS15371.1|
270	38	0.011	3	3	aldo keto reductase family 1 member B4	Function unknown	gi|674576243|emb|CDS37943.1|
271	24	0.015	3	3	ras protein rab 8b	Function unknown	gi|674571909|emb|CDS42331.1|
272	59	0.015	3	3	Synaptotagmin-14	-	gi|576700387|gb|EUB63909.1|
273	36	0.015	3	3	malate dehydrogenase	Energy production and conversion	gi|674563046|emb|CDS22680.1|
274	9	0.022	3	4	acyl coenzyme A binding protein	Metabolism	gi|674573492|emb|CDS40408.1|
275	323	0.011	3	3	talin	-	gi|674561411|emb|CDS24246.1|
276	26	0.015	3	3	Metallo-beta-lactamase domain-containing protein	-	gi|576694466|gb|EUB58053.1|
277	61	0.011	3	3	protein phosphatase 2a regulatory subunit	-	gi|674565771|emb|CDS20321.1|
278	31	0.015	3	3	aldo keto reductase family 1 member B4	Function unknown	gi|674564192|emb|CDS21190.1|
279	33	0.022	3	4	ATP synthase subunit gamma	Energy production and conversion	gi|576697467|gb|EUB61009.1|
280	97	0.015	3	3	Cysteinyl-tRNA synthetase, cytoplasmic	Transcription	gi|576701405|gb|EUB64924.1|
281	21	0.026	3	4	tegumental protein	-	gi|576695787|gb|EUB59351.1|
282	29	0.019	3	4	SCO cytochrome oxidase deficient protein 1	Function unknown	gi|674565543|emb|CDS20093.1|
283	68	0.015	3	3	nadp transhydrogenase	Energy production and conversion	gi|674566810|emb|CDS18157.1|
284	59	0.011	3	3	cytosolic purine 5' nucleotidase	-	gi|674562874|emb|CDS22508.1|
285	14	0.041	3	5	Profilin allergen	-	gi|674267056|emb|CDI97444.1|
286	83	0.015	3	3	Dipeptidyl peptidase 3	-	gi|576698676|gb|EUB62208.1|
287	30	0.015	3	3	N(G),N(G)-dimethylarginine dimethylaminohydrolase	Metabolism	gi|576699391|gb|EUB62919.1|

288	68	0.011	3	3	lamin dm0	-	gi|674567902|emb|CDS17015.1|
289	42	0.011	3	3	annexin	-	gi|674266071|emb|CDI98575.1|
290	17	0.022	3	5	ubiquitin conjugating enzyme	Post-translational modification	gi|532576613|gb|AGT98656.1|
291	24	0.0074	2	2	60S ribosomal protein L13a	Transcription	gi|576699387|gb|EUB62915.1|
292	48	0.015	2	2	Elongation factor 1-gamma	Post-translational modification	gi|576697550|gb|EUB61092.1|
293	42	0.0074	2	2	Alpha-tocopherol transfer protein-like protein	-	gi|576695576|gb|EUB59143.1|
294	44	0.0074	2	2	nardilysin, partial	Post-translational modification	gi|674561072|emb|CDS24623.1|
295	23	0.0074	2	2	60S ribosomal protein L13	Transcription	gi|576694227|gb|EUB57819.1|
296	35	0.0074	2	2	mitochondrial glutamate carrier protein	-	gi|555232805|emb|CDI70107.1|
297	29	0.0074	2	2	tegumental protein	-	gi|576693059|gb|EUB56683.1|
298	27	0.0074	2	2	aminoacylase 1	Metabolism	gi|674567544|emb|CDS18900.1|
299	75	0.0074	2	2	Dolichyl-diphosphooligosaccharide--protein glycosyltransferase subunit 2	-	gi|576692720|gb|EUB56357.1|
300	49	0.011	2	2	galactokinase	Metabolism	gi|674565167|emb|CDS20721.1|
301	20	0.011	2	3	FAD linked sulfhydryl oxidase ALR	Post-translational modification	gi|674570875|emb|CDS43499.1|
302	60	0.0074	2	2	puromycin sensitive aminopeptidase	Metabolism	gi|674560979|emb|CDS24674.1|
303	45	0.011	2	2	actin protein 3B	Structural/cytoskeletal proteins	gi|674580528|emb|CDS35468.1|
304	47	0.011	2	3	succinyl coenzyme A ligase ADP forming subunit beta	Energy production and conversion	gi|674576203|emb|CDS37901.1|
305	38	0.0074	2	2	vesicle associated membrane protein vamp	Signal transduction	gi|674564989|emb|CDS20538.1|
306	47	0.0074	2	2	Lysosomal aspartic protease	-	gi|576694392|gb|EUB57981.1|
307	22	0.0074	2	2	nascent polypeptide associated complex subunit	Transcription	gi|674571946|emb|CDS42368.1|
308	55	0.015	2	3	Protein disulfide-isomerase	Post-translational modification	gi|576692553|gb|EUB56196.1|
309	82	0.0074	2	2	procollagen lysine2 oxoglutarate 5 dioxygenase	-	gi|674576758|emb|CDS37197.1|
310	32	0.0074	2	2	Transcriptional activator protein Pur-alpha	-	gi|576701516|gb|EUB65035.1|
311	41	0.011	2	3	ribosomal protein L7-like protein Taenia asiatica]	Transcription	gi|124782929|gb|ABN14890.1|
312	17	0.011	2	2	60S ribosomal protein L27	Transcription	gi|576692810|gb|EUB56444.1|
313	18	0.019	2	3	Nucleoside diphosphate kinase A 2	Metabolism	gi|576695995|gb|EUB59555.1|
314	193	0.0074	2	2	intron binding protein aquarius	Transcription	gi|674570162|emb|CDS16230.1|
315	180	0.0074	2	2	collagen alpha 1(IV) chain Hymenolepis microstoma]	-	gi|674591161|emb|CDS29935.1|
316	13	0.011	2	3	Calmodulin	Structural/cytoskeletal proteins	gi|576700576|gb|EUB64098.1|

317	94	0.0074	2	2	Integrin beta-7	-	gi|576697707|gb|EUB61247.1|
318	51	0.0074	2	2	dihydrolipoyllysine residue acetyltransferase	Energy production and conversion	gi|674566907|emb|CDS18254.1|
319	30	0.011	2	3	hemicentin 1	-	gi|674576561|emb|CDS36997.1|
320	88	0.0074	2	2	Protein transport protein Sec23A	Signal transduction	gi|576697713|gb|EUB61253.1|
321	14	0.011	2	2	Four and a half LIM domains protein 3	-	gi|674266711|emb|CDI97590.1|
322	49	0.0074	2	2	expressed protein	-	gi|674561116|emb|CDS24544.1|
323	22	0.0074	2	2	Proteasome subunit beta type-2	Post-translational modification	gi|576700475|gb|EUB63997.1|
324	35	0.0074	2	2	actin related protein 2:3 complex subunit 1A:1B	Function unknown	gi|961439601|emb|CUT99097.1|
325	53	0.0074	2	2	succinate semialdehyde dehydrogenase	Energy production and conversion	gi|674561217|emb|CDS24488.1|
326	32	0.0074	2	2	Protein phosphatase 1 regulatory subunit	Function unknown	gi|576699343|gb|EUB62871.1|
327	10	0.011	2	2	Antigen B	-	gi|576692423|gb|EUB56071.1|
328	29	0.0074	2	2	Immunoglobulin domain containing protein	-	gi|674561326|emb|CDS24349.1|
329	67	0.0074	2	2	Leukotriene A-4 hydrolase	Metabolism	gi|576698893|gb|EUB62423.1|
330	16	0.015	2	2	Thioredoxin	Post-translational modification	gi|576693344|gb|EUB56960.1|
331	38	0.0074	2	2	Serine/threonine-protein phosphatase PP1-gamma catalytic subunit	Signal transduction	gi|576701522|gb|EUB65041.1|
332	531	0.011	2	2	dynein heavy chain	Structural/cytoskeletal proteins	gi|674569847|emb|CDS15915.1|
333	59	0.0074	2	2	pleckstrin y domain containing family A	-	gi|674560993|emb|CDS24688.1|
334	22	0.0074	2	2	expressed protein	Transcription	gi|674562273|emb|CDS23314.1|
335	31	0.011	2	2	Chloride intracellular channel protein 5	-	gi|576697068|gb|EUB60614.1|
336	19	0.0074	2	2	Ubiquitin-conjugating enzyme E2	Post-translational modification	gi|576695523|gb|EUB59091.1|
337	33	0.0074	2	2	LIN 24 Twenty four Like family member	-	gi|674266446|emb|CDI97898.1|
338	28	0.0074	2	2	Ndr	-	gi|674571386|emb|CDS41800.1|
339	20	0.011	2	3	glycogen phosphorylase	Metabolism	gi|674577298|emb|CDS37742.1|
340	12	0.011	2	2	FK506-binding protein-like protein	Post-translational modification	gi|194324935|gb|ACF49124.1|
341	11	0.0074	2	2	Dynein light chain 2, cytoplasmic	-	gi|576691997|gb|EUB55664.1|
342	70	0.0074	2	2	lamin	-	gi|674571622|emb|CDS42039.1|
343	16	0.011	2	2	Calcium binding protein	Structural/cytoskeletal proteins	gi|674576918|emb|CDS37358.1|
344	34	0.0074	2	2	Fascin-2	-	gi|576691449|gb|EUB55148.1|
345	69	0.0074	2	2	fimbrin Taenia asiatica]	Structural/cytoskeletal proteins	gi|124783291|gb|ABN14919.1|
346	44	0.011	2	2	GTP binding protein CG1354	Transcription	gi|674567571|emb|CDS18927.1|
347	80	0.0074	2	2	Armadillo type fold	-	gi|674265874|emb|CDI98378.1|
348	121	0.0074	2	2	dihydropyrimidine dehydrogenase NADP	Metabolism	gi|674568815|emb|CDS17936.1|

349	110	0.0074	2	2	SNase domain containing protein	Transcription	gi|674568828|emb|CDS17949.1|
350	42	0.0074	2	2	Erlin-1	Post-translational modification	gi|576691975|gb|EUB55643.1|
351	74	0.011	2	2	von Willebrand factor A domain-containing protein 5A	Function unknown	gi|576695423|gb|EUB58992.1|
352	40	0.0074	2	2	mitochondrial trans 2 enoyl coenzyme A reductase	Energy production and conversion	gi|674561473|emb|CDS24193.1|
353	8	0.011	2	3	immunogenic protein Ts76	-	gi|7339853|gb|AAF60976.1|
354	38	0.0074	2	2	dnaJ subfamily B	Post-translational modification	gi|961439579|emb|CUT99075.1|
355	17	0.011	2	3	Spectrin alpha actinin Hymenolepis microstomal	Structural/cytoskeletal proteins	gi|674591524|emb|CDS29622.1|
356	39	0.015	2	3	annexin	-	gi|674565627|emb|CDS20177.1|
357	50	0.011	2	2	NADH ubiquinone oxidoreductase 42 subunit	-	gi|674577975|emb|CDS36372.1|
358	51	0.0074	2	2	plastin 2	Structural/cytoskeletal proteins	gi|674573167|emb|CDS40079.1|
359	24	0.0074	2	2	60S ribosomal protein L15	Transcription	gi|576696309|gb|EUB59865.1|
360	75	0.0074	2	2	Metallophosphoesterase	Post-translational modification	gi|576694196|gb|EUB57789.1|
361	13	0.0074	2	2	60S ribosomal protein L23	Transcription	gi|576699448|gb|EUB62975.1|
362	25	0.011	2	3	Myophilin	Structural/cytoskeletal proteins	gi|961439407|emb|CUT98904.1|
363	26	0.011	2	3	proteasome prosome macropain subunit beta	Post-translational modification	gi|674565758|emb|CDS20308.1|
364	73	0.0074	2	2	alpha adducin	Metabolism	gi|674266887|emb|CDI97275.1|
365	17	0.015	2	2	NADH dehydrogenase ubiquinone] 1 beta subcomplex subunit	-	gi|576697957|gb|EUB61494.1|
366	8	0.0074	2	2	40S ribosomal protein S28	Transcription	gi|576698176|gb|EUB61712.1|
367	34	0.015	2	2	mitochondrial carrier 2	-	gi|674566620|emb|CDS19720.1|
368	38	0.0074	2	2	Dehydrogenase:reductase SDR family	Metabolism	gi|674577428|emb|CDS36875.1|
369	12	0.015	2	2	expressed conserved protein	-	gi|674577450|emb|CDS36897.1|
370	46	0.015	2	4	calcium binding protein calreticulin precursor	-	gi|14029538|gb|AAK52725.1|
371	26	0.019	2	3	ES1 protein mitochondrial	Metabolism	gi|674568144|emb|CDS17259.1|
372	25	0.0074	2	2	NADH dehydrogenase ubiquinone iron sulfur	Energy production and conversion	gi|674561353|emb|CDS24291.1|
373	56	0.0074	2	2	protein disulfide isomerase A3	Post-translational modification	gi|674572082|emb|CDS42507.1|
374	48	0.0074	2	2	mitogen activated protein kinase	Signal transduction	gi|674574349|emb|CDS41278.1|
375	47	0.011	2	2	Enolase	Metabolism	gi|576691849|gb|EUB55524.1|
376	20	0.0074	2	2	ATP synthase subunit d	-	gi|674575042|emb|CDS39544.1|
377	79	0.0074	2	2	Long-chain-fatty-acid--CoA ligase 5	Metabolism	gi|576699113|gb|EUB62642.1|
378	97	0.0074	2	2	Lon protease	Post-translational modification	gi|576696096|gb|EUB59654.1|

379	84	0.019	2	4	hypothetical protein	Function unknown	gi|21912588|emb|CAD21549.1|
380	13	0.0074	2	2	expressed protein	-	gi|674571192|emb|CDS43186.1|
381	86	0.0074	2	2	hypothetical transcript	-	gi|961440318|emb|CDS37307.2|
382	10	0.015	2	2	immunogenic protein	-	gi|674577101|emb|CDS37545.1|
383	28	0.0074	2	2	EF-1, partial	Transcription	gi|7578954|gb|AAF64192.1|
384	17	0.0074	2	2	60S ribosomal protein L14	Transcription	gi|576698217|gb|EUB61752.1|
385	337	0.0074	2	2	twitchin	Signal transduction	gi|674566777|emb|CDS18123.1|
386	239	0.0074	2	2	otoferlin	-	gi|674565985|emb|CDS19081.1|
387	63	0.0074	2	2	T-complex protein 1 subunit epsilon	Post-translational modification	gi|576697091|gb|EUB60637.1|
388	26	0.011	2	2	ATP synthase subunit O	Energy production and conversion	gi|576698231|gb|EUB61766.1|
389	92	0.0074	2	2	U6 snRNA-associated Sm-like protein LSm8	-	gi|576695080|gb|EUB58655.1|
390	43	0.0074	2	2	WD40 repeat and fyve domain containing protein	-	gi|674561477|emb|CDS24197.1|
391	19	0.0074	2	2	hypothetical protein EGR_00893	-	gi|576700828|gb|EUB64349.1|
392	36	0.0074	2	2	Nuclear migration protein nudC	-	gi|576696811|gb|EUB60360.1|
393	35	0.0074	2	2	Ts5 protein	Transcription	gi|170783723|gb|ACB37346.1|
394	58	0.0074	2	2	Calnexin	-	gi|576694840|gb|EUB58420.1|
395	73	0.0074	2	2	cationic amino acid transporter 4	Metabolism	gi|674568117|emb|CDS17231.1|
396	11	0.011	2	2	heat shock protein	Post-translational modification	gi|576691723|gb|EUB55405.1|
397	21	0.011	2	2	MACRO domain containing protein 2	Function unknown	gi|674571215|emb|CDS43209.1|
398	19	0.015	2	4	titin	-	gi|961439545|emb|CUT99041.1|
399	14	0.0074	2	2	NADH dehydrogenase ubiquinone 1 beta	-	gi|674561702|emb|CDS24017.1|
400	88	0.0074	2	2	microtubule associated protein 1A	-	gi|674561041|emb|CDS24648.1|
401	54	0.0074	2	2	titin	-	gi|674567709|emb|CDS16820.1|
402	26	0.0074	2	2	Ras-related protein Rab-2A	Function unknown	gi|576699325|gb|EUB62853.1|
403	29	0.0074	2	2	Endophilin-B1	-	gi|576695776|gb|EUB59340.1|
404	59	0.011	2	2	T complex protein 1 subunit eta	Post-translational modification	gi|674562117|emb|CDS23628.1|
405	33	0.011	2	2	aldo keto reductase family 1 member B4	Function unknown	gi|674266305|emb|CDI97752.1|
406	50	0.022	2	2	Tubulin beta chain	Structural/cytoskeletal proteins	gi|576695709|gb|EUB59274.1|
407	65	0.0074	2	2	Fatty-acid amide hydrolase	Transcription	gi|576697203|gb|EUB60748.1|
408	22	0.0074	2	2	Protein archease	Function unknown	gi|674266531|emb|CDI97985.1|
